# An injectable hydrogel combining medicine and matrix with anti-inflammatory and pro-angiogenic properties for potential treatment of myocardial infarction

**DOI:** 10.1093/rb/rbad036

**Published:** 2023-04-19

**Authors:** Jiayin Feng, Min Xing, Wenhao Qian, Jiajun Qiu, Xuanyong Liu

**Affiliations:** State Key Laboratory of High Performance Ceramics and Superfine Microstructure, Shanghai Institute of Ceramics, Chinese Academy of Sciences, Shanghai 200050, China; Center of Materials Science and Optoelectronics Engineering, University of Chinese Academy of Sciences, Beijing 100049, China; Shanghai Xuhui District Dental Center, Shanghai 200032, China; Shanghai Xuhui District Dental Center, Shanghai 200032, China; State Key Laboratory of High Performance Ceramics and Superfine Microstructure, Shanghai Institute of Ceramics, Chinese Academy of Sciences, Shanghai 200050, China; State Key Laboratory of High Performance Ceramics and Superfine Microstructure, Shanghai Institute of Ceramics, Chinese Academy of Sciences, Shanghai 200050, China; Center of Materials Science and Optoelectronics Engineering, University of Chinese Academy of Sciences, Beijing 100049, China; Shanghai Engineering Research Center of Nano-Biomaterials and Regenerative Medicine, College of Biological Science and Medical Engineering, Donghua University, Shanghai 201620, China

**Keywords:** mesoporous silica, puerarin, hydrogels, angiogenesis, myocardial repair

## Abstract

One of the main illnesses that put people’s health in jeopardy is myocardial infarction (MI). After MI, damaged or dead cells set off an initial inflammatory response that thins the ventricle wall and degrades the extracellular matrix. At the same time, the ischemia and hypoxic conditions resulting from MI lead to significant capillary obstruction and rupture, impairing cardiac function and reducing blood flow to the heart. Therefore, attenuating the initial inflammatory response and promoting angiogenesis are very important for the treatment of MI. Here, to reduce inflammation and promote angiogenesis in infarcted area, we report a new kind of injectable hydrogel composed of puerarin and chitosan via *in situ* self-assembly with simultaneous delivery of mesoporous silica nanoparticles (CHP@Si) for myocardial repair. On the one hand, puerarin degraded from CHP@Si hydrogel modulated the inflammatory response via inhibiting M1-type polarization of macrophages and expression of pro-inflammatory factors. On the other hand, silica ions and puerarin released from CHP@Si hydrogel showed synergistic activity to improve the cell viability, migration and angiogenic gene expression of HUVECs in both conventional and oxygen/glucose-deprived environments. It suggests that this multifunctional injectable CHP@Si hydrogel with good biocompatibility may be an appropriate candidate as a bioactive material for myocardial repair post-MI.

## Graphical Abstract

**Figure:**
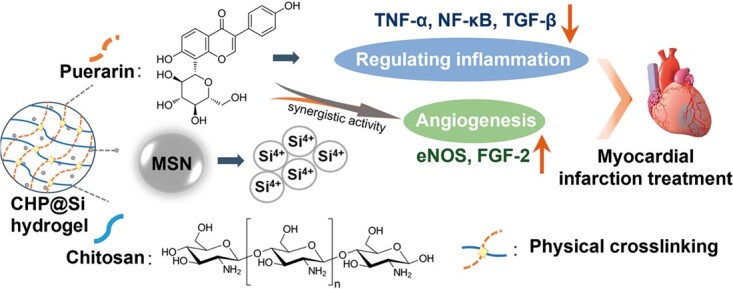

## Introduction

Cardiovascular diseases pose a great risk to human health. Among them, myocardial infarction (MI), which has the highest incidence, is myocardial necrosis resulting from hypoxia and ischemia in the coronary arteries due to atherosclerosis and thrombosis. After MI, the myocardial microenvironment has been greatly altered. The ischemia and hypoxia lead to myocardial cell death [1], and apoptotic cardiomyocytes trigger myocardial ischemia and local inflammatory response, resulting in myocardial fibrosis and scar formation [2], which can lead to heart failure and ventricular remodeling in severe cases [3]. Additionally, MI causes the continuous degradation of the extracellular matrix, which thins ventricular wall and enlarges ventricles. Pharmacological and interventional therapies are two primary categories of the current treatment approaches [4]. However, these current clinical treatments have major limitations, such as contraindications, unpleasant effects or high complications, which have limited their practical implementation. Heart transplantation is currently the treatment that can fundamentally restore normal heart function for patients, but it is extremely limited by the rarity of heart donors, the high invasiveness of the procedure and the expensive cost [5, 6].

Numerous studies have demonstrated the efficacy of injectable hydrogels in treating myocardial injury [7–9]. On the one hand, it is very flexible, extremely biocompatible and simple to modify. It can be injected into the body through the endocardium, epicardium or coronary arteries, eliminating invasive surgical procedures and significantly lowering the additional harm surgery to the body [10]. On the other hand, hydrogels have a porous structure and can be utilized as carriers to load various functional molecules such as cell growth factors [8], drugs [11], nanoparticles [12] or mRNA [13], safeguarding these functional molecules while enhancing utilization.

Chitosan-based hydrogel is applied frequently in the area of myocardial repair due to its superior biocompatibility [14–16]. Chitosan macromolecular chain contains a high proportion of hydroxyl and amino groups, which makes it easy to be chemically modified or combined with various bioactive molecules or drugs [17, 18]. Chitosan hydrogel could be produced by chemical or physical cross-linking. The former is mostly processed by covalent cross-linking of chitosan with cross-linking agents such as formaldehyde [19], glutaraldehyde [20] and glycol diglycerides [21]. While the latter relies on non-covalent interactions such as intermolecular hydrophobic interactions [22], hydrogen bonding [23] and electrostatic interactions [24] to form gels. However, chemically cross-linked hydrogels tend to be biotoxic, while physically cross-linked hydrogels, although have better biocompatible and less toxic, mostly with poor stability and mechanical properties, which limit their further applications in clinical practice. Therefore, it is crucial to overcome these deficiencies and ensure that chitosan-based hydrogels have superior biosafety and stability.

Puerarin is a monomeric component of Chinese medicine that is derived from the Pueraria lobata plant. It has the ability to promote myocardial blood circulation, prevent thrombosis and atherosclerosis, and has a wide-ranging application in treatment of cardiovascular diseases [25, 26]. Puerarin could contribute to the repair of damaged myocardium by modulating the inflammatory response, inhibiting pro-inflammatory expression and anti-oxidative damage [27, 28]. However, the water solubility and the lipid solubility of puerarin are not very well, which may affect human absorption, and produce some side effects [25, 29]. Interestingly, puerarin dissolved via high temperature or cosolvent can form supramolecular hydrogels by self-assembly [30], it not only solves the problem of poor solubility of puerarin but also achieves the self-delivery of active components. However, the hydrogel formed by the self-assembly of puerarin has poor mechanical properties and cannot be used directly for clinical use. Recent studies have developed chitosan@puerarin composite hydrogels for the treatment of skin wounds using an herbal grinding method [31] and as a potential sustained and controlled deliverer of chlorinated berberine hydrate [32] and gene-targeted drugs for intraocular melanoma while being antibacterial [33]. It implies that puerarin can be interacted with chitosan by self-assembly to form hydrogels with unique characteristics of interactive network structure, stronger mechanical properties and ‘combination of medicine and matrix’. However, most current research focuses on the antibacterial property of composite hydrogels or their use as carriers but does not discuss the chitosan puerarin composite hydrogels on potential applications of myocardial repair through the regulation of inflammation.

Since the rupture of a large number of capillaries after MI further exacerbates the inadequate blood supply to the heart, angiogenesis is crucial to promote the repair of infarcted myocardium [34]. Bioactive ions with functional effects are important in the area of tissue engineering as they have clinical application possibilities: more stability, lower cost and potentially higher safety than synthetic proteins, growth factors or DNA recombinant technologies [35]. Substantial research efforts have shown silica-containing biomaterials can promote angiogenesis [36–38], such as calcium silicate bioceramics that release silicate ions to stimulate angiogenesis by upregulating VEGF in the endothelial cells and activating vascular endothelial growth factor receptor (KDR) [39]. Chang *et al*. [40] reported ‘ion therapy’ as a new therapeutic strategy in MI, they found a remarkable increase in cardiomyocyte viability, increased angiogenesis and improved cardiac function after silicone-rich ion extracts were injected intravenously into mice with MI. It indicates that silicon ions have great potential as bioactive ions for application in myocardial repair. It has been suggested that mesoporous silica nanoparticles (MSNs) could undergo degradation and release silica ions [41–45]. In contrast to the complex degradation products of silicate-based bioactive glasses, MSNs only release silicon ions, excluding the possibility that the positive effects produced by bioactive glasses may be the result of various ionic synergisms [46, 47].

Based on the above considerations, we innovatively designed a new injectable hydrogel with the matrix itself that has tissue repair-inducing activity. Hydrogel fabricated by *in situ* self-assembly of puerarin and chitosan and delivering mesoporous silica (CHP@Si) for myocardial repair, which may achieve mechanical reinforcement, anti-inflammatory and pro-angiogenesis properties simultaneously. The physical characteristics of CHP@Si hydrogel, including surface morphologies, gelation time, mechanical property, degradation rate and ions release behavior, were investigated to reveal the applicability in clinical treatment. Furthermore, the effects of puerarin and silicon ions released from the composite hydrogel on the inflammatory response and synergistic activity in stimulating angiogenesis and maintaining of HUVECs viability in both conventional and oxygen/glucose-deprived (OGD) environments were confirmed *in vitro*. Our research findings suggest that this multifunctional injectable CHP@Si hydrogel may be an appropriate candidate as a bioactive material for MI repair.

## Materials and methods

### Preparation and characterization of MSNs

MSNs were fabricated following the previously reported literature [46]. Briefly, 0.91 g of cetyltrimethylammonium bromide (Sinopharm Group, China) and 1.5 g of ammonium fluoride (NH_4_F, Sinopharm Group, China) were dissolved in 250 ml of ultrapure water at 80°C and then 4.5 ml of tetraethoxysilane (Sinopharm Group, China) was slowly added with stirring. Stirred for 2 h and let the solution stand overnight. The precipitates were captured by centrifugation (8000 rpm, 15 min), washed three times with ultrapure water, then three times with alcohol. The dried powder was calcined at 600°C in air with heating rate of 1°C·min^−1^ for 6 h.

The microstructure, release and degradation of MSNs from hydrogel were investigated by Tecnai G2 F20 transmission electron microscopy (TEM, Shimadzu, Japan) with an acceleration voltage of 120 kV. The release and degradation test of MSNs from hydrogel was performed as follows: 50 mg of CHP@Si hydrogels were immersed in 1 ml phosphate buffer saline (PBS) at 37°C for 1, 3, 7 and 14 days, and the supernatant was collected after centrifugation at the specified time points and supplemented with 1 ml of fresh PBS. MSNs in the supernatant were observed using TEM. The phase composition of MSNs was analyzed using X-ray Diffractomator (XRD, Rigaku, Japan). The particle size distribution of MSNs was tested with the multi-angle particle size analyzer (90Plus PALS, Brookhaven, USA).

N_2_ adsorption–desorption experiment was performed at 77.3K to attain the mesopore size distribution of MSNs using the automatic four-station specific surface pore size analyzer (Quadrasorb SI, Quantachrome, USA).

### Preparation of CHP@Si composite hydrogels

Adding 100 mg of low viscosity chitosan and 100 mg of medium viscosity chitosan (CS, ≥95% deacetylation degree, Aladdin, China) to 10 ml of 0.6 vol.% glacial acetic acid (Sinopharm Group, China) aqueous solution and stirred until the chitosan was completely dissolved. Dissolving 0.1 g of β-glycerol phosphate disodium salt pentahydrate (β-GP, TCL, Shanghai, China) in 0.5 ml of ultrapure water. Subsequently, adding 17.5 mg of puerarin (PUE, Sinopharm, China) to 0.5 ml of β-GP solution and stirred at 85°C until puerarin was completely dissolved, then adding the β-GP-PUE solution drop by drop to 2.5 ml of chitosan solution. Thirty-five milligrams of the above-synthesized MSNs were introduced into the CS-β-GP-PUE solution and stirred until completely mixed. Subsequently, 0.5 ml of 2% (w/v) aqueous solution of hydroxyethylcellulose (HEC, TCL, Shanghai, China) was introduced to the above mixture and stirred until the gel was formed, the corresponding samples were named as CHP@Si (CS/HEC/β-GP-PUE/MSNs). Similar method was used to prepare CH(CS/HEC), CHP(CS/HEC/β-GP-PUE) and CH@Si (CS/HEC/MSNs) hydrogels.

### Characterization of CHP@Si composite hydrogels

The cross-sectional morphology of hydrogels was observed by scanning electron microscopy (SEM, S-3400 N, Hitachi, Japan) with a working voltage of 5 kV. The hydrogels were freeze-dried and then quenched in liquid nitrogen to obtain cross-sections, followed by morphology observation with SEM after spraying platinum. FTIR spectra was detected in the range of 4000–400 cm^−1^ with FTIR spectrometer (Tensor 27, Bruker, Germany).

Dynamic rheology of hydrogels was measured with the rheometer (MCR301, Anton Paar, Austria). The variation of storage modulus (*G*′) and loss modulus (*G*″) with frequency at 25°C, 1% strain and the variation of *G*′ and *G*″ with strain at 25°C, 1 Hz were recorded.

To investigate the degradation rate, the hydrogels were submerged in PBS (Gibco Invitrogen Inc) solution for 1, 3, 7 and 14 days. Weighing the hydrogel at fixed time. The degradation rate of hydrogel was calculated by Equation (1) where *W*_0_ represents the original mass of hydrogel, and *W_t_* indicates the mass of hydrogel at different times.


(1) $$\mathrm{Hydrogel}\;\mathrm{degradation}\;\mathrm{rate}=\frac{W_{0}-W_{t}}{W_{0}}$$

To study the release behavior of Si ions from the hydrogel, the hydrogels were submerged in PBS solution (50 mg/ml). The PBS solution was replaced at the specific time points and measurement of Si ion concentration in PBS solution was performed by inductively coupled plasma atomic emission spectrometer (ICP-AES, Prodigy Plus, Leeman, USA).

### Cell culture

L929 cells, HUVECs and RAW264.7 were obtained from the Cell Bank of the CAS, Shanghai. L929 cells were cultured with α-MEM (Gibco, USA) and 10% fetal bovine serum (FBS, Gibco, USA), and 1% double antibodies (penicillin and streptomycin, Hyclone, USA). HUVECs were cultured with ECM (Sciencell, USA) complete medium, and RAW264.7 cells were cultured with DMEM (Gibco, USA) and 15% FBS and 1% double antibodies. These cells were incubated at 37°C in the incubator containing 5% CO_2_ and 95% relative humidity.

### Preparation of hydrogels extracts

To prepare extracts of hydrogels, 500 mg of hydrogels sterilized by UV irradiation for 2 h were submerged in 10 ml of α-MEM, DMEM, or ECM at 37°C for 24 h. Then the extracts of hydrogels were filtered with 0.22 μm sterile filter (PALL 4612, USA).

### Live/dead cell staining

Assessment of the effects of CH, CHP, CH@Si and CHP@Si hydrogels on cell viability was carried out using the live/dead cell staining kits (Biovision, USA). To be specific, L929 cells were introduced into 24-well plates (5 × 10^4^ cells per well) and incubated for 12 h. Then replaced the medium with α-MEM extracts of CH, CHP, CH@Si and CHP@Si hydrogels and continued to incubate. After 24 h, discarded all media and washed the cells with PBS two times. Added 300 μl of PBS solution containing calcein-AM and propidium iodide to every well and incubated at 37°C for 30 min. After that, fluorescence photographs of live/dead cells staining of L929 were taken with the fluorescence microscope (Olympus, Japan). In the same way, fluorescence photographs of live/dead cell staining of HUVECs were obtained.

### Cell viability assessment

For cell viability assay, L929 cells and HUVECs were respectively seeded in a 24-well plate (5 × 10^4^ cells per well) and cultured for 12 h. The medium was then changed with fresh cell culture medium, CH, CHP, CH@Si and CHP@Si hydrogels extracts and continued incubation for 24 h. 500 μl of FBS-free medium containing 10% alamarBlue (Invitrogen™, Thermo, USA) was added to every well and keep incubating cells for 2 h. AlamarBlue is reduced to a strongly fluorescent compound after entering living cells, and its fluorescence intensity is proportional to cell viability. Reduced alamarBlue fluorescence intensity was examined with the Microplate Reader (Cytation 5, BioTek, USA). Cell viability was calculated by Equation (2)


(2) $$\mathrm{Cell}\;\mathrm{viability}=\frac{{\mathrm{OD}}_{\mathrm{SPL}}-{\mathrm{OD}}_{0}}{{\mathrm{OD}}_{\mathrm{Con}}-{\mathrm{OD}}_{0}}\times 100\%$$

Respectively, OD_SPL_, OD_0_ and OD_Con_ represent the absorbance of the hydrogel groups, the 10% alamarBlue group without hydrogels and cells, and the control group.

### RAW 264.7 macrophage polarization *in vitro*

To explore the modulating effect of hydrogels on inflammation, firstly, hydrogels containing different concentrations of puerarin were synthesized as follows: 0.6 vol.% aqueous glacial acetic acid solution was mixed with 100 mg of low viscosity chitosan and 100 mg of medium viscosity chitosan to obtain 10 ml of chitosan solution. Then dissolving 0.1 g of β-glycerophosphate disodium salt (β-GP) in 0.5 ml of ultrapure water. Subsequently, adding 17.5 mg of PUE to 0.5 ml of β-GP solution and stirred at 85°C until PUE was completely dissolved, followed by transferring drop by drop into 2.5 ml of chitosan solution. At last, the above mixture was added with 0.5 ml of 2% (w/v) HEC aqueous solution and stirred until the gel was formed, the corresponding hydrogel was named P5 (the concentration of puerarin in the hydrogel was 5 mg/ml). Similar method was used to prepare P0, P10 and P20 samples.

Next, the extracts of hydrogels containing different concentrations of puerarin were prepared: Under 37°C for 24 h, 500 mg of UV sterilized hydrogels were submerged in 10 ml DMEM. Then the extracts of hydrogels were filtered with 0.22 μm sterile filter (PALL 4612, USA). Afterward, RAW264.7 were seeded in a six-well plate (1.0 × 10^6^ cells per well). After 24 h of incubation, replaced the culture medium with hydrogels extract containing 100 ng/ml LPS. The polarization morphology of RAW 264.7 macrophage was recorded by microscopy under bright field after 24 h.

### Tube formation assay

One hundred twenty microliters of matrix gel (356234, Corning, USA) was pipetted into every well of a 48-well plate at 4°C. The plate was then placed at 37°C for 30 min until the matrix gel turned into the gel state. Subsequently, HUVECs were planted on the matrix gel (3 × 10^4^ cells per well), and the medium was replaced with the extract of CHP@Si0, CHP@Si5, CHP@Si10 and CHP@Si20 hydrogels after 4 h when the cells fully adhered. Observation and photographic recording of tube formation of HUVECs was performed using fluorescence microscopy in bright field mode after 12 h. Randomly selected three areas in each well for photography.

### Scratch assay

HUVECs were introduced into a 6-well plate (1.5 × 10^5^ cells per well). When the cell confluence reached 80% or more, draw a straight line at the bottom of each well with a 200 μL pipette tip. Then, every well was rinsed three times with PBS and the medium was changed with ECM extracts of CH, CHP, CH@Si and CHP@Si hydrogels. After 0, 12 and 24 h, cell migration was recorded by photographing in bright-field mode with fluorescence microscopy. Three areas in each well were randomly selected for observation. Migration rate of cells was calculated as Equation (3) where *L*_0_ represents the scratch width of 0 h, and *L*_t_ represents the scratch width of different times.


$$\text{Cell migration rate}=\frac{L_{0}-L_{t}}{L_{0}}\times 100\%$$

### Oxygen/glucose-deprived model of HUVECs *in vitro*

Simulation of damage to HUVECs by ischemic and hypoxic conditions in MI was performed using the OGD model. The hypoxic conditions were established as previously described [48]. HUVECs were introduced into a 96-well plate (3 × 10^3^ cells per well) and a 6-well plate (1.5 × 10^5^ cells per well) at 37°C for 24 h. After that, replaced the medium with glucose/serum-free DMEM (Gibco), and placed inside a sealed container that contains an AnaeroPack (MGC, Japan). After culturing in OGD conditions for 4 h, cell culture plates were removed from the sealed containers to terminate hypoxia, and replacing the glucose/serum-free DMEM with CH, CHP, CH@Si and CHP@Si hydrogel extracts containing serum and glucose. After continuing the culture for 1 day, detection of cell viability in 96-well plate, scratch assay and qRT-PCR were performed on cells cultured in six-well plate to evaluate cell migration ability and expression of pro-angiogenic genes.

Similarly, introducing 3 × 10^3^ HUVECs per well in a 96-well plate for 24 h. Then, replacing the culture media with glucose/serum-free DMEM extracts of CH, CHP, CH@Si and CHP@Si hydrogels. Subsequently, the cells were placed in the hypoxic environment and culture for 4 h. Finally, alamarBlue was used to assess the protective efficacy of the hydrogel on HUVECs by testing cell viability under OGD conditions.

### Quantitative real-time polymerase chain reaction

Introduced 1.0 × 10^6^ RAW 264.7 per well into a six-well plate for 24 h, and then changed the culture medium with the DMEM extracts of hydrogels containing 100 ng/ml of LPS. Continued incubation for 24 h, discarding the medium and washing with PBS two times for every well, then 1 ml of Trizol (Invitrogen, Thermo, USA) reagent was added to each well to extract total cellular RNA.

Similarly, introducing 1.5 × 10^5^ HUVECs per well into a six-well plate for 24 h, the incubation was continued for 24 h after replacing the medium with the ECM extract of hydrogels. Discarding the medium and washing with PBS two times for every well, then 1 ml of Trizol reagent was added to each well to extract total cellular RNA.

After extraction of total cellular RNA with Trizol reagent, reverse transcription of mRNA to cDNA was carried out using the Transcriptor First Strand cDNA Synthesis Kit (Roche). Follow the instructions to use SYBR Green Master Mix and qPCR analysis was carried out with the Roche LightCycler 480 System. GAPDH was used as a normalization control for all genes. Supplementary Table S1 lists the primer sequences used in this study.

### Statistical analysis

Data analysis was performed using GraphPad Prism 8.3.0. The unpaired *t*-test, one-way ANOVA and two-way ANOVA were used to determine significance of the data. *P < 0.05, **P < 0.01, ***P < 0.001 and ****P < 0.0001.

## Results and discussion

### Modulation of inflammation by Puerarin *in vitro*

**Figure 1.**
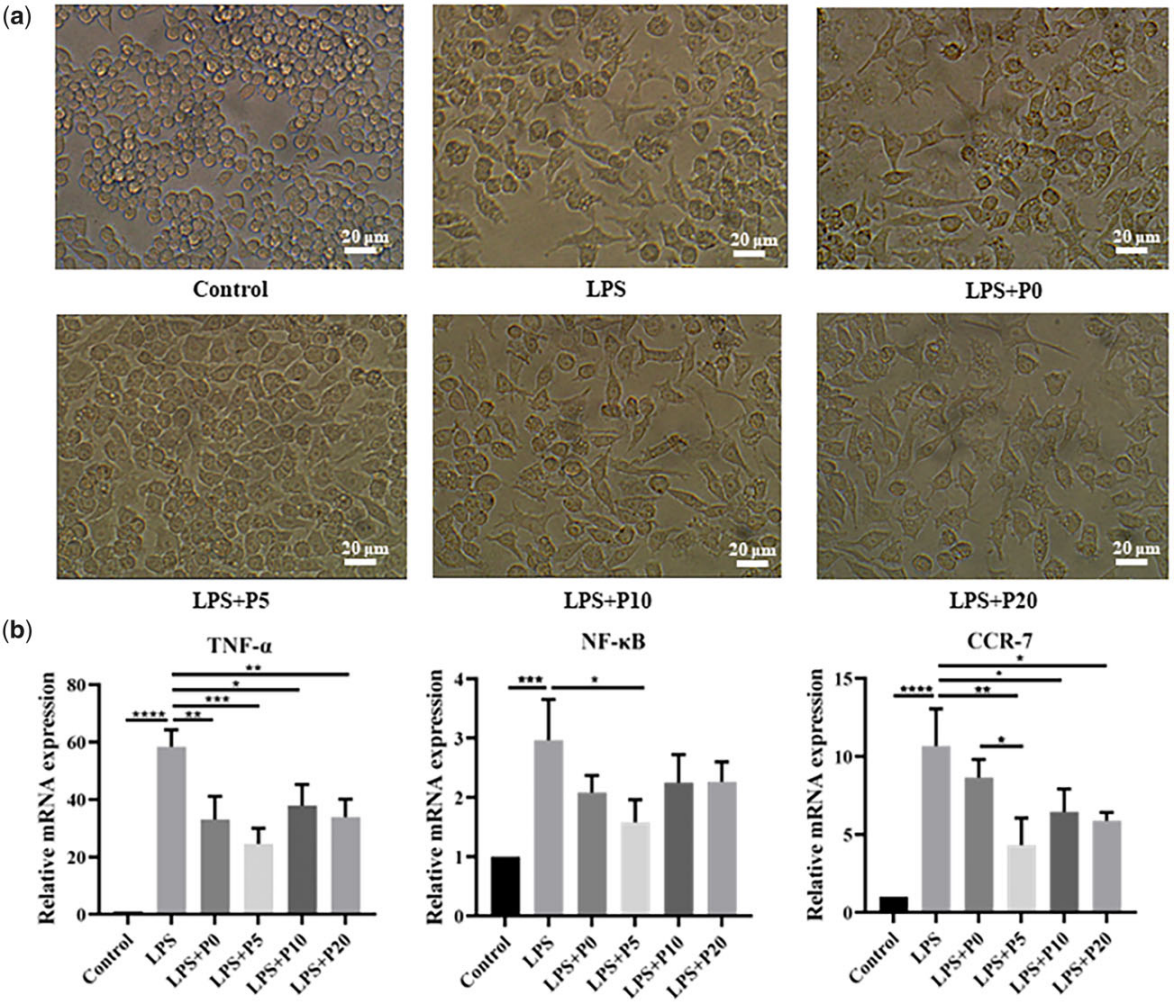
Effects of hydrogels with puerarin in different concentrations on the polarization morphology of macrophages *in vitro*. (**a**) RAW 264.7 macrophage morphology after being treated with LPS or LPS with hydrogels containing different concentrations of puerarin. Scale bars = 20 μm. (**b**) qRT-PCR quantification of TNF-α, NF-κB and CCR-7 mRNA expression. *P <* *0.05, **P <* *0.01, ***P < 0.001 and ****P <* *0.0001.

Based on the function and inflammatory factor secretion, macrophages are divided into two subsets, M1-type macrophages and M2-type macrophages. M1-type macrophages cause necrosis and apoptosis of cardiomyocytes and play a major role in promoting the development of inflammation [49]. To research the regulatory effect of puerarin on inflammation and determine the appropriate concentration of puerarin in hydrogels, LPS was used to induce M1-type polarization of RAW 264.7 macrophages and followed by co-culture with hydrogels containing different concentrations of puerarin (0, 5, 10 and 20 mg/ml). The results show that the cell morphology of macrophages becomes flattened and extended pseudopods after stimulation with LPS (Fig. 1a), which indicates the successful polarity of M1-type macrophages [50]. However, cell morphology becomes round and cell size turns small after co-culture with hydrogels containing different concentrations of puerarin, especially for the LPS + P5 group. Moreover, the expression of relevant inflammatory genes in macrophages was examined by qRT-PCR and the experimental results are presented in Fig. 1b. Relative expression of pro-inflammatory genes including TNF-α, NF-κB and CCR7 is remarkably increased after LPS stimulation. However, the relative expression of these pro-inflammatory genes is differentially reduced in the hydrogels group. These results demonstrate that puerarin has the ability to inhibit MI-type polarization of macrophages and reduce the expressions of pro-inflammatory genes.

Hydrogels with 5 mg/ml of puerarin showed the best effect for inhibiting pro-inflammatory gene expression, compared to that 10 and 20 mg/ml. The concentration of puerarin has a great influence on its biological effects. It has been found that a high concentration of puerarin with 100 mM caused cytotoxicity to HaCaT cells [51]. The cell viability of HUVECs was reduced after 24 h by the treatment of 500 μM puerarin, and puerarin at concentration of 50–200 μM does not show obvious cytotoxicity to HUVECs [52]. These suggest that a high concentration of puerarin could inhibit cell activity.

Therefore, we deduce that the hydrogels with puerarin concentrations of 10 and 20 mg/ml released higher concentrations of puerarin, which may cause some negative effects on cell viability. In contrast, the amount of puerarin released from the hydrogel with the puerarin concentration of 5 mg/ml was appropriate and therefore showed the optimal effect in suppressing pro-inflammatory gene expression. Thus, the P5 group with the puerarin concentration of 5 mg/ml was selected for the follow-up experiments.

### Characterization and degradation behavior of MSNs

**Figure 2.**
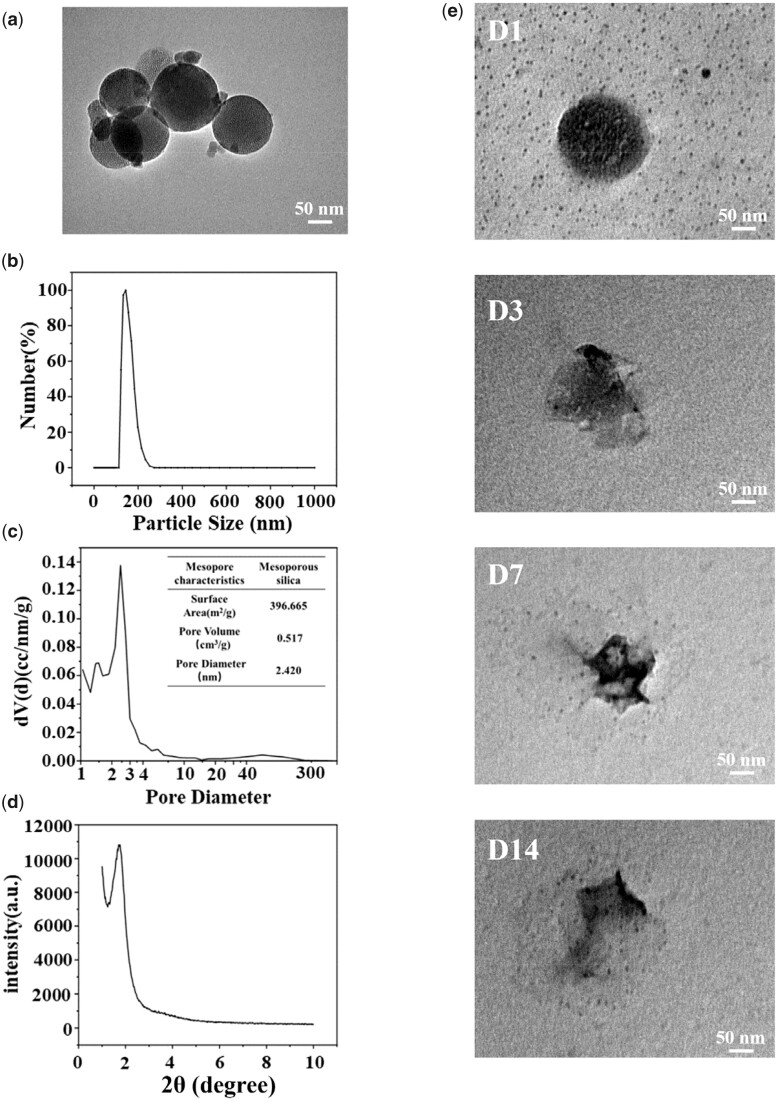
Surface characterization of mesoporous silica and its degradation process in hydrogels. (**a**) Surface morphology of mesoporous silica observed by TEM. (**b**) Particle size, (**c**) pore diameter and (**d**) XRD spectra of MSNs. (**e**) Morphological changes of mesoporous silica in hydrogels submerged in PBS for 1, 3, 7 and 14 days. Scale bars = 50 nm.

Figure 2a presents the TEM surface morphology of MSNs. The particle size distribution of MSNs is around 170 nm detected by the dynamic light scattering technique (Fig. 2b). As indicated in Fig. 2c, the surface area, pore volume and pore diameter of MSNs are 396.665 m^2^/g, 0.517 cm^3^/g and 2.42 nm, respectively. The small-angle X-ray diffraction result shows an obvious diffraction peak at 2θ = 2°, which suggests that MSNs have ordered mesostructure (Fig. 2d). Moreover, the degradation process of MSNs was also investigated, and the result is presented in Fig. 2e. From the TEM images, it can be found that MSNs show a gradual degradation trend with time, which implies that silicon ions can be released through degradation to achieve tissue repair effect.

### Pro-angiogenic property of MSNs

**Figure 3.**
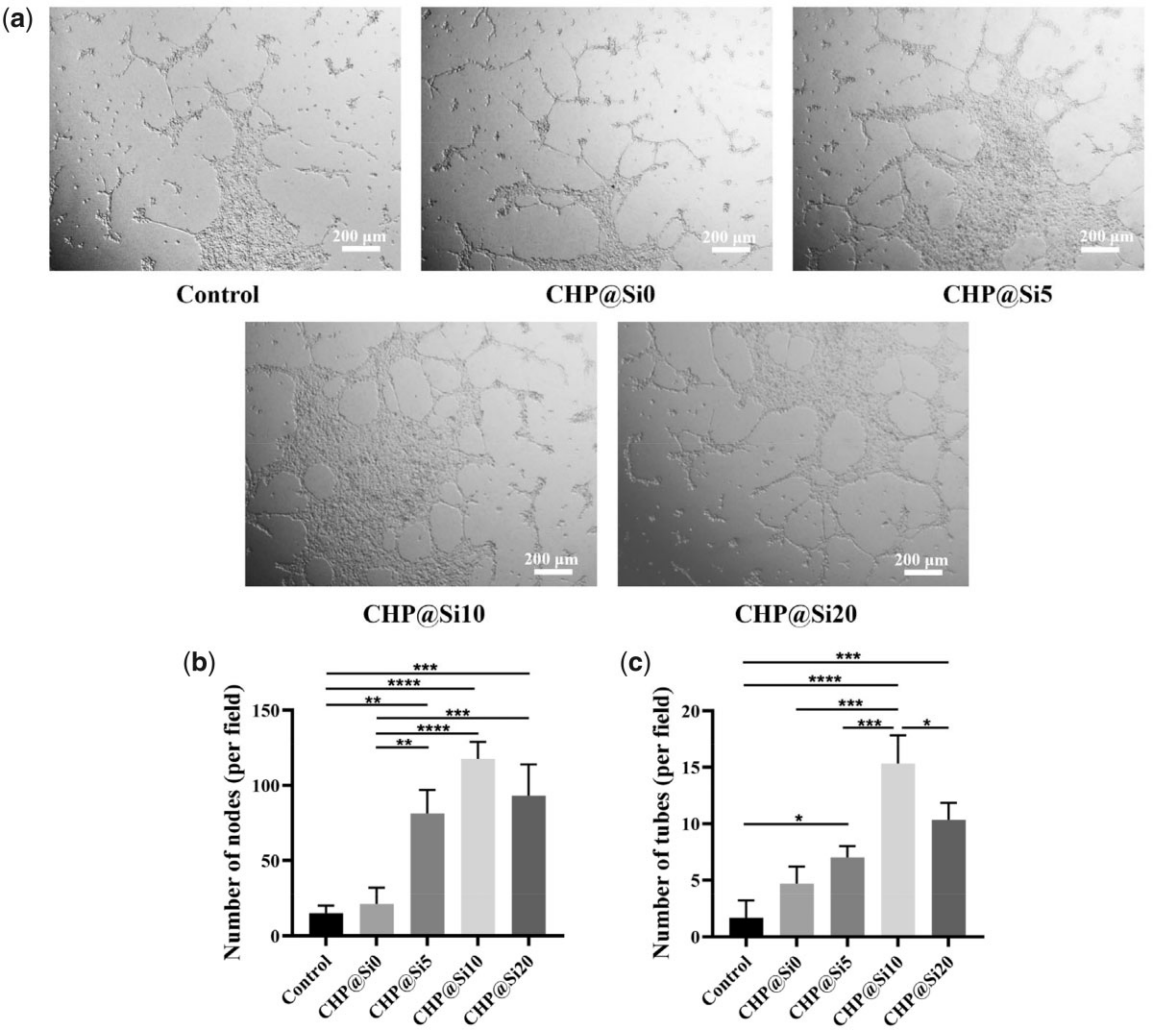
Effects of hydrogels containing different concentrations of MSNs on vascularization ability of HUVECs. (**a**) Tube-forming ability of HUVECs treated by hydrogels containing different concentrations of mesoporous silica, scale bars = 200 μm. (**b**) Number of tube nodes. (**c**) Number of tubes. *P <* *0.05, **P <* *0.01, ***P < 0.001 and ****P <* *0.0001.

To study the pro-angiogenic property of MSNs and confirm the appropriate composite ratio of MSNs in hydrogels, a tube formation assay of HUVECs was performed by co-culture of HUVECs and hydrogels containing 0, 5, 10 and 20 mg/ml of MSNs. The results show that Si ions can facilitate HUVECs to form tubular polygonal network structures *in vitro* (Fig. 3a), and the tube-forming ability is correlated with the concentration of MSNs, which is concentration-dependent at the range of 0–10 mg/ml. However, 20 mg/ml of MSNs inhibited the formation of tubes (Fig. 3b and c). This is because MSNs can cause some damage to the cells when the concentration is too high [53, 54]. Therefore, hydrogel containing 10 mg/ml of MSNs was used as the follow-up experimental groups.

### Characterization of CHP@Si hydrogel

**Figure 4.**
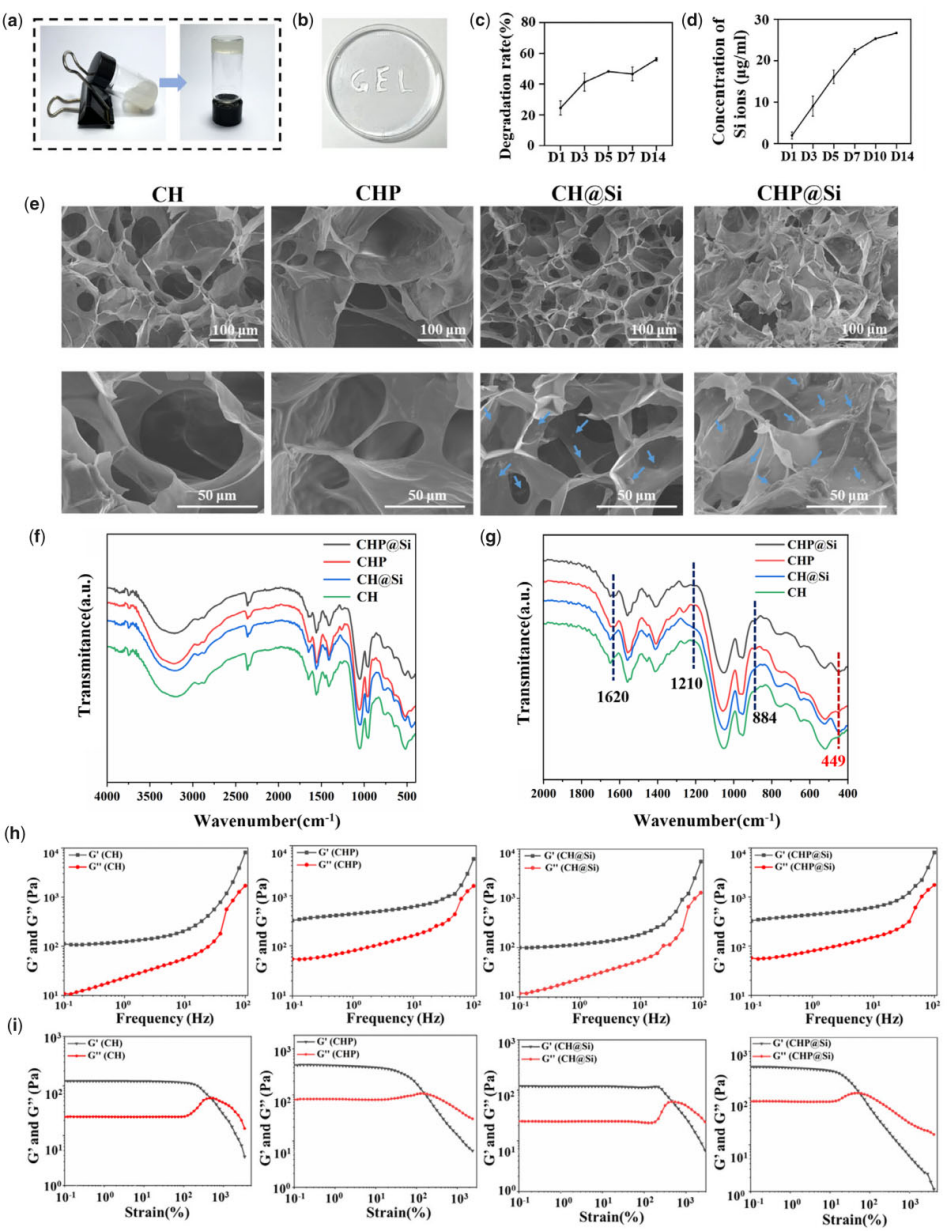
Physicochemical properties of hydrogels. (**a**) The sol–gel transition of CHP@Si hydrogel. (**b**) Injectability of CHP@Si hydrogel. (**c**) The degradation rate of CHP@Si hydrogel. (**d**) Concentration of Si ions released from CHP@Si hydrogel. (**e**) SEM image of CH, CHP, CH@Si and CHP@Si hydrogels. (**f**) FTIR spectra of CH, CHP, CH@Si and CHP@Si hydrogels in the range of 4000–400 cm^−1^ and (**g**) 2000–400 cm^−1^. (**h**) Variation of storage modulus (*G*′) and loss modulus (*G*″) of hydrogel with frequency. (**i**) Variation of *G*′ and *G*″ of hydrogel with strain.

After determining the effective contents of puerarin and MSNs, we constructed the CHP@Si hydrogel. The sol–gel process of CHP@Si hydrogel is presented in Fig. 4a. In the soluble state, CHP@Si retains the liquid state and can flow freely, while the CHP@Si hydrogel made by the above synthetic scheme does not flow or deform after 1 min of inversion after gel formation. It proves that the CHP@Si hydrogel has been successfully formed. Puerarin can be dissolved in hot water and self-assembled to form supramolecular hydrogels after cooling [30]. Yuan *et al*. co-heated puerarin with chitosan solution and used self-assembly of puerarin with chitosan molecular chain entanglement to form composite hydrogels [32]. However, chitosan is a polymeric polysaccharide and may undergo denaturation or inactivation at high temperatures. It was found that when β-GP was used as the solvent, puerarin could be completely dissolved after heating, and the solution remained clear and transparent after cooling down without precipitation of puerarin. This is because when puerarin is heated and dissolved, the glycerol group of β-GP and water molecules form binding water by hydrogen bonding around the puerarin molecule chain, and then form a water shell layer around the puerarin molecules and prevent the molecules of puerarin from entangling with each other and precipitating out of the solution after cooling. In the present work, β-GP was used as a solvent to dissolve puerarin at high temperature and then mixed with chitosan solution at room temperature to form hydrogels by self-assembly, which not only avoided the denaturation of chitosan molecules but also ensured that the puerarin in the hydrogels existed in a completely dissolved and uniformly dispersed form.

Figure 4b shows the CHP@Si hydrogel can be injected into a ‘GEL’ shape by 1 ml syringe and maintains its original gel state after injection, which indicates that CHP@Si hydrogel has good injectability and it is convenient to inject into the site of MI through minimally invasive surgery.

It has been shown that chitosan hydrogels can provide support for damaged myocardium and facilitate myocardial repair [55, 56]. CHP@Si hydrogel degraded by 60% after 14 days of immersion in PBS (Fig. 4c), which shows a suitable degradation rate for long-term action at the infarct site and to supply stable support for the damaged heart. The concentration of silica ions released in CHP@Si hydrogel was tested by ICP, the result is presented in Fig. 4d. Silica ions can be released from the hydrogel until Day 14. This indicates that the CHP@Si hydrogel can achieve a long-term slow release of silica ions.

Cross-sectional morphology of hydrogels was observed by SEM, and all hydrogels show porous 3D network structure. Since the MSNs surface is negatively charged (Supplementary Fig. S1) and the chitosan molecules are positively charged. They are closely bonded through electrostatic interaction [57], and MSNs tightly bound on network structure can be seen from CH@Si and CHP@Si hydrogels, as shown in Fig. 4e.

Figure 4f and g shows the FTIR spectra ranging from 4000 to 400 and 2000 to 400 cm^−1^ of hydrogels, respectively. In Fig. 4f, the broad absorption bands at 3800–3100 cm^−1^ correspond to the -OH and -NH_2_ groups observed from all the samples, which results in the formation of a high amount of hydrogen bonds in the hydrogels system. In Fig. 4g, compared to CH and CH@Si, new peaks at 1620, 1210 and 884 cm^−1^ can be seen from CHP and CHP@Si, which correspond to absorption peaks of PUE at 1629, 1231 and 897 cm^−1^ (Supplementary Fig. S2). The newly appeared absorption peaks in the CHP and CHP@Si hydrogels shift to lower wave numbers compared to PUE, which indicates the presence of PUE in hydrogels and that the formation of hydrogen bond stabilization structure between CS and PUE influences the location of PUE absorption peaks in hydrogels. In addition, new absorption peaks are observed at 449 cm^−1^ in CH@Si and CHP@Si hydrogels, which corresponds to the absorption peak of MSNs at 449 cm^−1^ (Supplementary Fig. S3). Figure 4h and i shows the dynamic rheological test results. *G*′ is the energy storage modulus, and *G*″ is the loss modulus, when the *G*′ > *G*″ proves the formation of hydrogels. It is found that *G*′ and *G*″ of CHP and CHP@Si hydrogels are significantly increased after the addition of PUE compared with CH and CH@Si hydrogels, which is ascribed to the hydrogen bonds between PUE and chitosan molecular chains improving the mechanical strength of hydrogels.

### Biocompatibility evaluation

**Figure 5.**
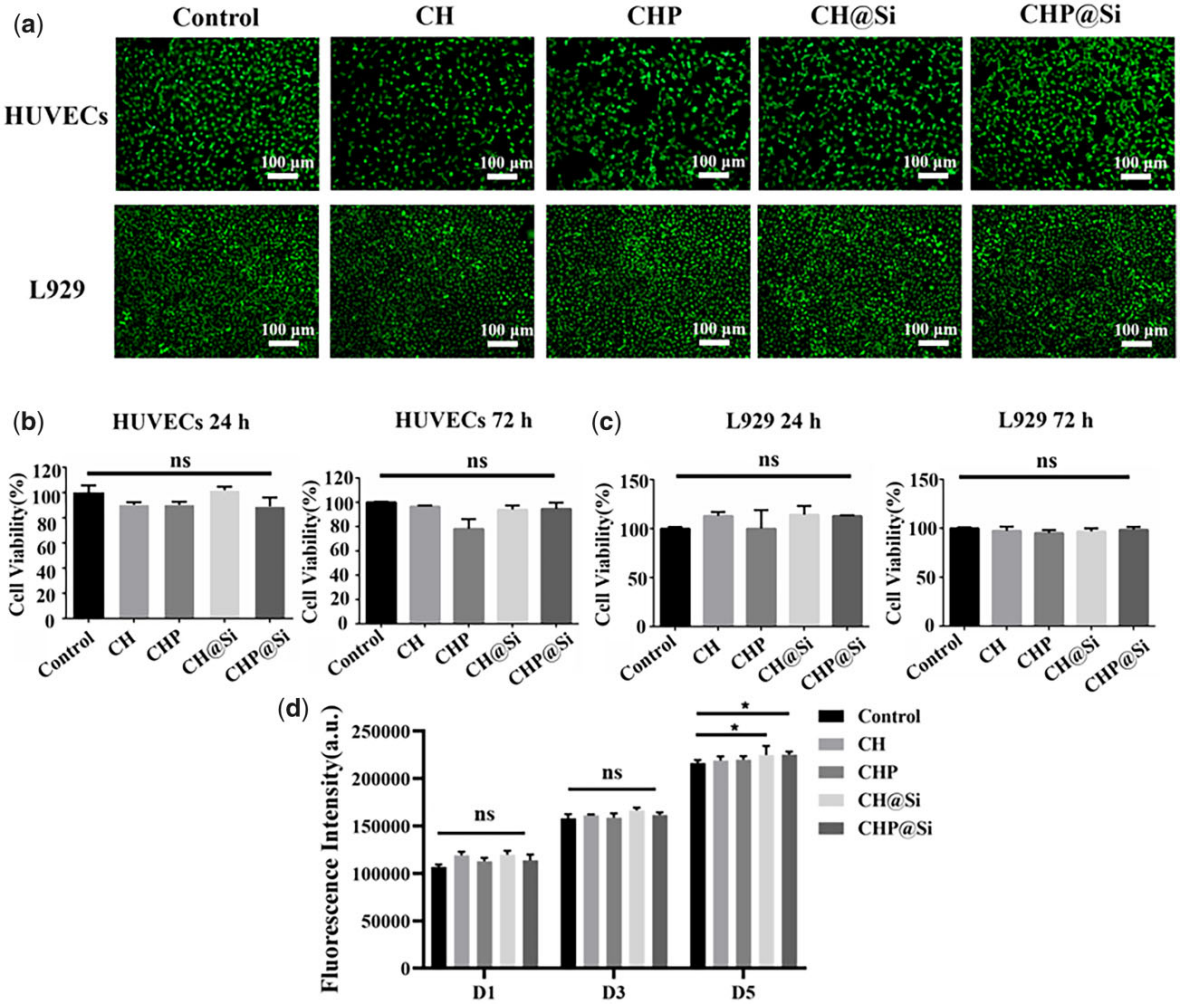
Cytocompatibility of hydrogels. (**a**) Fluorescence images of live/dead cell staining of HUVECs and L929 cells treated with CH, CHP, CH@Si and CHP@Si hydrogels for 24 h, scale bars = 100 μm. (**b**) Cell viability of HUVECs treated with CH, CHP, CH@Si and CHP@Si hydrogels for 24 h and 72 h. (**c**) Cell viability of L929 cells cultured with CH, CHP, CH@Si and CHP@Si hydrogels for 24 h and 72 h. (**d**) Cell viability of HUVECs treated with CH, CHP, CH@Si and CHP@Si hydrogels for 1, 3 and 5 days by detecting the fluorescence intensity of reduced alamarBlue. *P < 0.05.

The biocompatibility of biomaterials is the primarily concerned issue. Thus, cell viability assay and live/dead cell staining of L929 and HUVECs were performed to evaluate the biocompatibility of CHP@Si hydrogel. After live/dead staining, live cells appear green while dead cells appear red, and almost no dead cells are observed in Fig. 5a, indicating that none of hydrogels were significantly cytotoxic. Figure 5b and c shows that cell viability was above 80% after treatment with CHP@Si hydrogel for 24 and 72 h, and there was no remarkable difference between the hydrogels. It indicates that these hydrogels have good biocompatibility.

After culturing HUVCEs with hydrogels for 1, 3 and 5 days, cell viability was tested with alamarBlue. In the first 3 days, cell viability treated with hydrogels was not remarkably different from the control group, while on the fifth day, the cell viability of CH@Si and CHP@Si hydrogels was higher than control and other hydrogels groups. It suggests that Si ions can promote the proliferation of HUVECs.

### Modulation of inflammation by hydrogels *in vitro*

In cardiac remodeling after MI, apoptosis-induced inflammatory cascade response recruits immune cells to clear apoptotic cells, while releasing growth factors that promote massive fibroblast proliferation and exacerbate scar formation [58]. Numerous studies have shown that suppression of excessive inflammation in the early stage of MI facilitates the reduction of cardiac fibrosis and promotes myocardial repair [59, 60]. To study the influence of hydrogels on the modulation of inflammation, different groups were designed to examine the effects of hydrogels containing no puerarin and MSN (CH), only puerarin (CHP), only MSN (CH@Si) and both puerarin and MSN (CHP@Si) on cells.

It was found that CH hydrogel (without PUE and MSNs) further exacerbated the inflammatory response relative to the LPS group. Chitosan with a deacetylation degree of more than 95% is not soluble in water, but soluble in many dilute inorganic or organic acids such as formic acid, glacial acetic acid etc. CH hydrogel was formed by dissolving chitosan in 6‰ glacial acetic acid and then mixed with 2% HEC. Therefore, CH hydrogel has weak acidic pH value. As the optimal pH for cell culture is generally ∼7.2, the weak acidic pH has a certain irritating effect on cells. Low environmental pH could lead to increased TNF-α synthesis and NO production in inflamed tissues [61]. That may be why the LPS+CH group with weak acidic pH increases the inflammatory response to some extent.

**Figure 6.**
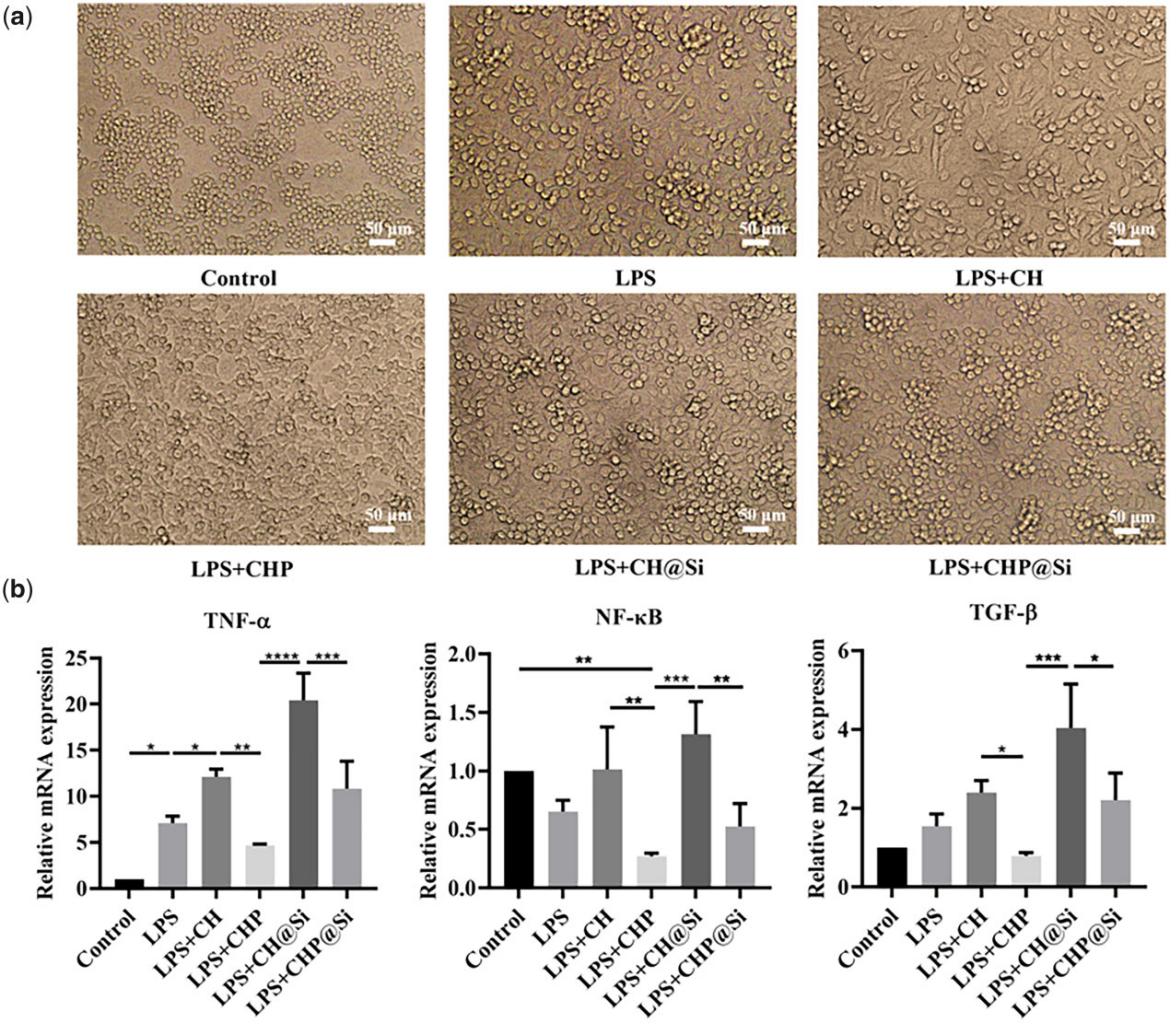
Effects of CH, CHP, CH@Si and CHP@Si hydrogels on macrophage polarization morphology and inflammatory gene expression *in vitro*. (**a**) Surface morphology of RAW 264.7 cells treated with LPS or LPS with CH, CHP, CH@Si and CHP@Si hydrogels. Scale bars = 50 μm. (**b**) qRT-PCR quantification of TNF-α, NF-κB and TGF-β mRNA expression. *P <* *0.05, **P <* *0.01, ***P < 0.001 and ****P <* *0.0001.

When the hydrogel contained only PUE (CHP, pH of 7.0), macrophage polarization was inhibited, which cell morphology was close to M0 type (Fig. 6a) and the expression of pro-inflammatory factors including TNF-α, NF-κB and TGF-β was remarkably reduced (Fig. 6b).

However, the relative expression level of pro-inflammatory factors was again upregulated by the hydrogel containing MSNs alone (CH@Si) and was higher than CH, which could be due to two reasons: the pH of CH@Si hydrogel is close to that of CH hydrogel and its lower pH has a stimulating effect on cells; CH@Si hydrogel is free of puerarin and therefore have a looser structure. High concentrations of silica ions are suddenly released at an early stage, which has a stimulating effect on macrophages [56, 62, 63].

When PUE and MSNs coexisted (CHP@Si), the expression of inflammatory genes in macrophages showed a decreasing trend. The addition of puerarin not only improves the pH of the hydrogel but also delays the release of silica ions. The above results suggest that CHP@Si hydrogel can regulate the inflammatory response and inhibit the expression of pro-inflammatory genes which is closely dependent on the effect of PUE.

### Effects of hydrogels on the proliferation, migration and angiogenic activity of HUVECs

**Figure 7.**
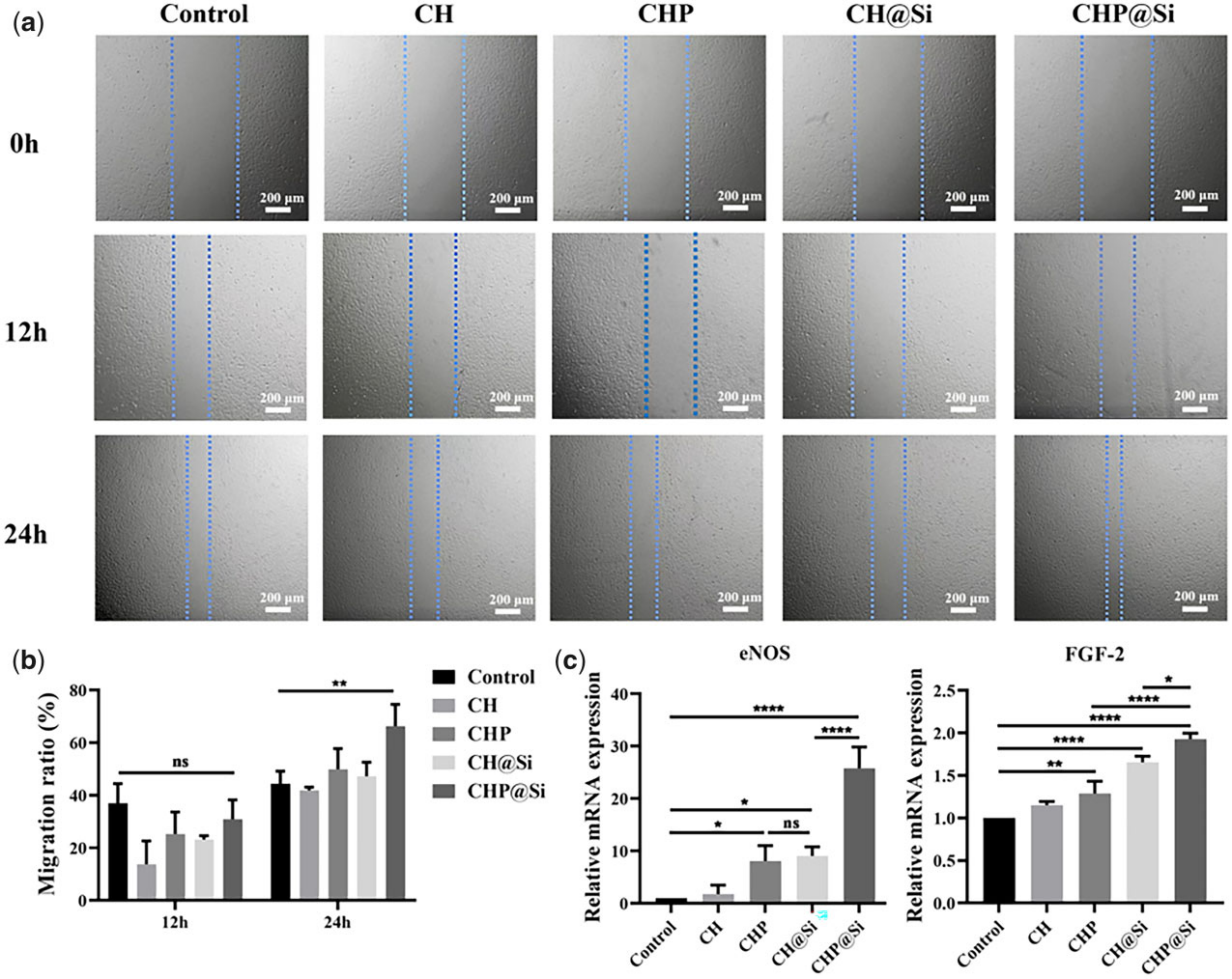
Effects of CH, CHP, CH@Si and CHP@Si hydrogels on cell proliferation, migration, and gene expression of HUVECs. (**a**) Migration of HUVECs treated with CH, CHP, CH@Si and CHP@Si hydrogels at 0, 12 and 24 h. Scale bars = 200 μm. (**b**) Cell migration rate. (**c**) qRT-PCR quantification of eNOS and FGF-2 mRNA expression. *P <* *0.05, **P <* *0.01 and ****P <* *0.0001.

The ischemic and hypoxic environment after infarction can cause massive capillary blockage and rupture, exacerbating inadequate blood supply to the heart and resulting in enlargement of the infarcted area. Restoration of blood flow to the infarcted tissue reconstruction could supply adequate oxygen and nutrition to the cells in the infarcted area. Therefore, promoting angiogenesis in the infarcted region is another key issue in myocardial repair [64]. As shown in Fig. 7a and b, CHP@Si hydrogel notably promoted the migration of HUVECs and increased the expression of angiogenic genes eNOS and FGF-2 (Fig. 7c). The expression of eNOS and FGF-2 was enhanced when either PUE or MSNs was present alone. When both PUE and MSNs were present, the expressions of eNOS and FGF-2 were significantly elevated, suggesting a pro-angiogenic synergistic effect between PUE and MSNs. This is because, after the onset of MI, fibrosis and ventricular dilatation caused by inflammation lead to poor angiogenesis resulting in a lack of nutrients and oxygen to myocardial tissue. Puerarin is able to regulate inflammation and improve the infarct microenvironment, while having a vasodilatation effect and increasing blood flow [65], thus promoting myocardial blood flow reconstruction. While the pro-angiogenic ability of silica ions has been confirmed in previous experiments and literature [66, 67]. The coexistence of PUE and MSNs can further promote angiogenesis and enhance the repair effect.

### Protective and restorative effect of hydrogels on HUVECs under OGD conditions

**Figure 8.**
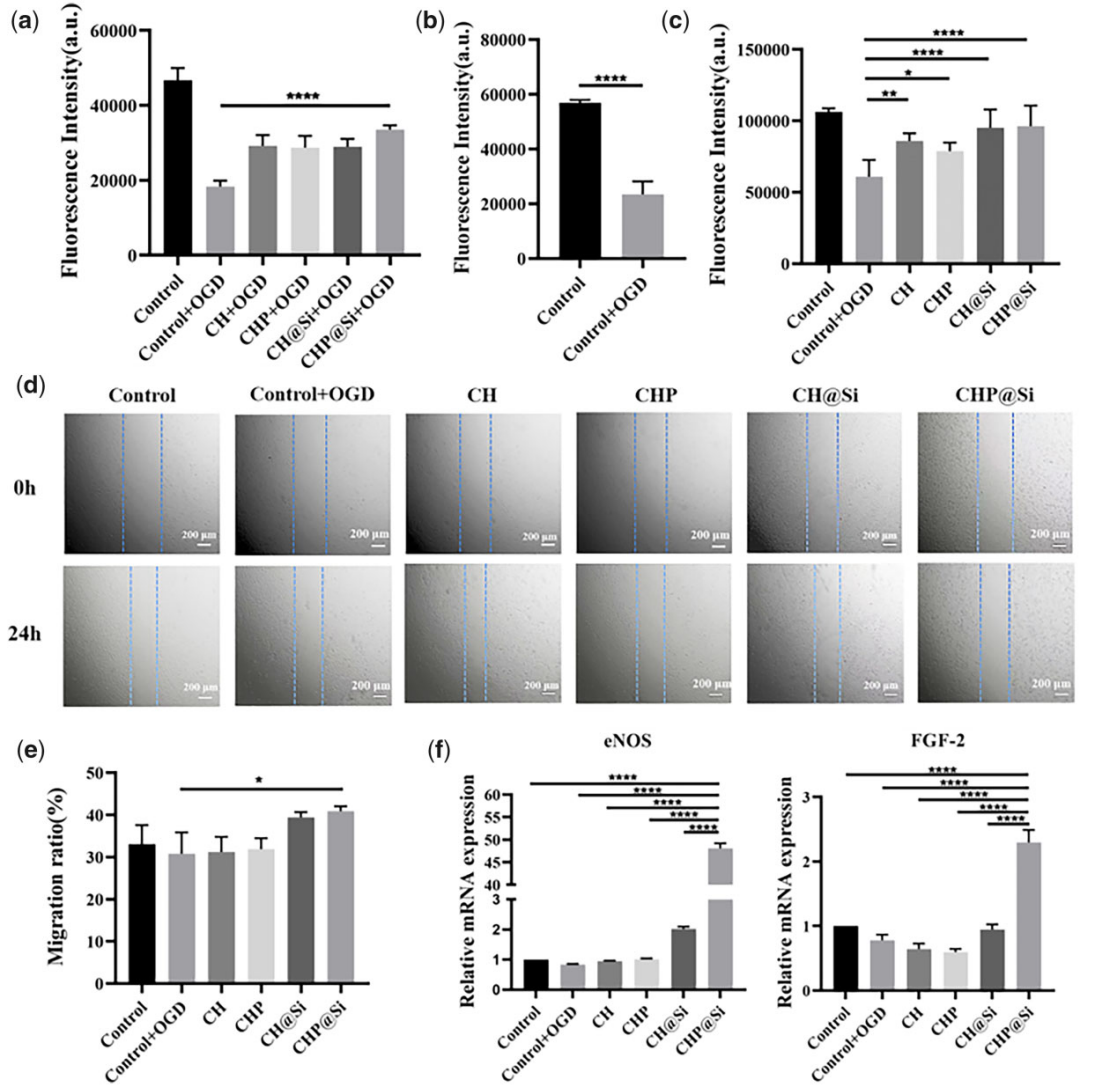
Effects of CH, CHP, CH@Si and CHP@Si hydrogels on cell viability, migration and gene expression of HUVECs under OGD. (**a**) The viability of HUVECs treated with CH, CHP, CH@Si and CHP@Si hydrogels under OGD conditions for 4 h. (**b**) The viability of HUVECs cultured under OGD for 4 h. (**c**) The viability of HUVECs co-cultured with CH, CHP, CH@Si and CHP@Si hydrogels for 1 day after cultured under OGD conditions for 4 h. (**d**) Cell migration of HUVECs co-cultured with CH, CHP, CH@Si and CHP@Si hydrogels for 1 day after cultured under OGD conditions for 4 h. Scale bars = 200 μm. (**e**) Cell migration rate. (**f**) qRT-PCR quantification of eNOS and FGF-2 mRNA expression. *P <* *0.05, **P <* *0.01 and ****P < 0.0001.

MI leads to vascular endothelial cells apoptosis due to the deficient blood and oxygen supply. Protection and renewal of the vascular endothelial cells activity under OGD conditions is essential to effectively re-establish blood flow and has been identified as one of the feasible treatments for MI [15]. In this work, the OGD model *in vitro* was employed to simulate injury to HUVECs under MI microenvironment *in vivo*. After being cultured with hydrogels under OGD conditions for 4 h, cell viability was detected by alamarBlue and it was found that HUVECs treated with CHP@Si hydrogel retained strong cell viability (Fig. 8a), which indicates that the hydrogels have a protective effect on HUVECs under OGD conditions. Cell viability was significantly decreased after 4 h of OGD culture (Fig. 8b). After 4 h of OGD treatment of HUVECs, the medium was changed with the extracts of each group of hydrogels and cultured for 24 h. The repair effect of hydrogels on cells damaged by OGD was verified by cell viability assay, cell migration assay, and qRT-PCR. The cells treated with CHP@Si hydrogel have the highest cell viability (Fig. 8c) and cell migration rate (Fig. 8d and e), and CHP@Si hydrogel could significantly increase the expression of pro-angiogenic factors eNOS and FGF-2 (Fig. 8f). These findings indicate that CHP@Si hydrogel can not only protect endothelial cells to reduce the damage caused by OGD, but also relieve the oxidative stress damage after reoxygenation of cells, restore cell vitality and promote cell migration and angiogenesis.

## Conclusion

In this study, we constructed a chitosan/HEC/puerarin/MSNs (CHP@Si) composite hydrogel with unique features of unification of medicine and matrix and as a carrier for the continuous release of bioactive silicon ions. The addition of puerarin could enhance the mechanical properties and modulate the inflammatory response, inhibiting M1-type polarization of macrophages and production of pro-inflammatory factors. Moreover, it was found that silica ions generated by the degradation of MSNs could promote the formation of the tubular network structure of HUVECs *in vitro*. In addition, there was a synergistic effect between MSNs and PUE which could significantly enhance the migration ability of HUVECs and upregulate the expression of angiogenic-related genes in both conventional and OGD environments. All these findings suggest that injectable CHP@Si hydrogel with anti-inflammatory and pro-angiogenesis properties could be an excellent candidate for MI repair materials and has potential for use in clinical treatment.

## Supplementary Material

rbad036_Supplementary_Data
