# Supplementary material for: An injectable hydrogel combining medicine and matrix with anti-inflammatory and pro-angiogenic properties for potential treatment of myocardial infarction
Source: Regen Biomater. 2023 Apr 19;10:rbad036. doi: 10.1093/rb/rbad036 (PMC10159687; doi:10.1093/rb/rbad036)
Supplement: rbad036_Supplementary_Data [file rbad036_supplementary_data.docx]

**Supplementary Data**

**An injectable hydrogel combining medicine and matrix with anti-inflammatory and pro-angiogenic properties for potential treatment of myocardial infarction**

Jiayin Feng ^a, b, 1^, Min Xing ^c, 1^, Wenhao Qian ^c^, Jiajun Qiu ^a,*^, Xuanyong Liu ^a, b, d,*^

^a^ State Key Laboratory of High Performance Ceramics and Superfine Microstructure, Shanghai Institute of Ceramics, Chinese Academy of Sciences, Shanghai 200050, China

^b^ Center of Materials Science and Optoelectronics Engineering, University of Chinese Academy of Sciences, Beijing 100049, China

^c^ Shanghai Xuhui District Dental Center, Shanghai 200032, China

^d^ Shanghai Engineering Research Center of Nano-Biomaterials and Regenerative Medicine, College of Biological Science and Medical Engineering Donghua University, 2999 North Renmin Road, Shanghai 201620, China

^1^ These authors contributed equally to this work.

* Correspondence: xyliu@mail.sic.ac.cn (X. Liu), qiujiajun@mail.sic.ac.cn (J. Qiu)


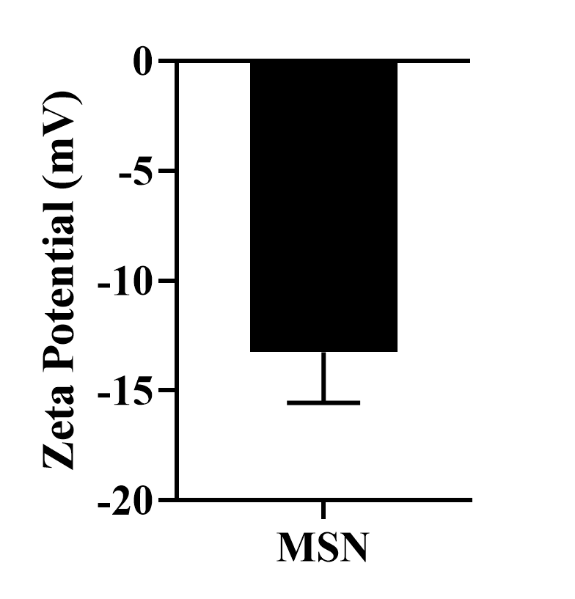


**Figure S1.** Zeta potential value of MSN.

The mesoporous silica nanoparticles (MSNs) were dispersed in ultrapure water and homogenized by ultrasound, and then surface zeta potential of the MSNs was measured by the zeta potential particle size analyzer (90 Plus, Brookhaven Instruments, USA). The result shows that the surface of MSNs is negatively charged (Figure S1).


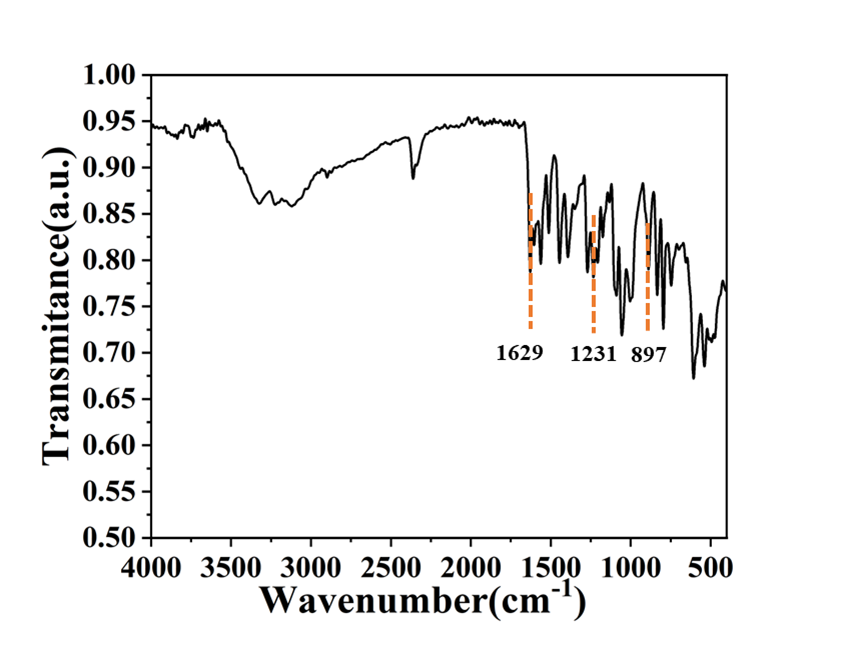
**Figure S2.** FTIR spectra of puerarin in the range of 4000-400 cm^-1^.

The FTIR spectra of puerarin was measured from 4000 to 400 cm^-1^ by FTIR spectrometer. Puerarin shows clear absorption peaks at 1629, 1231, and 897 cm^-1^ (Figure S2).


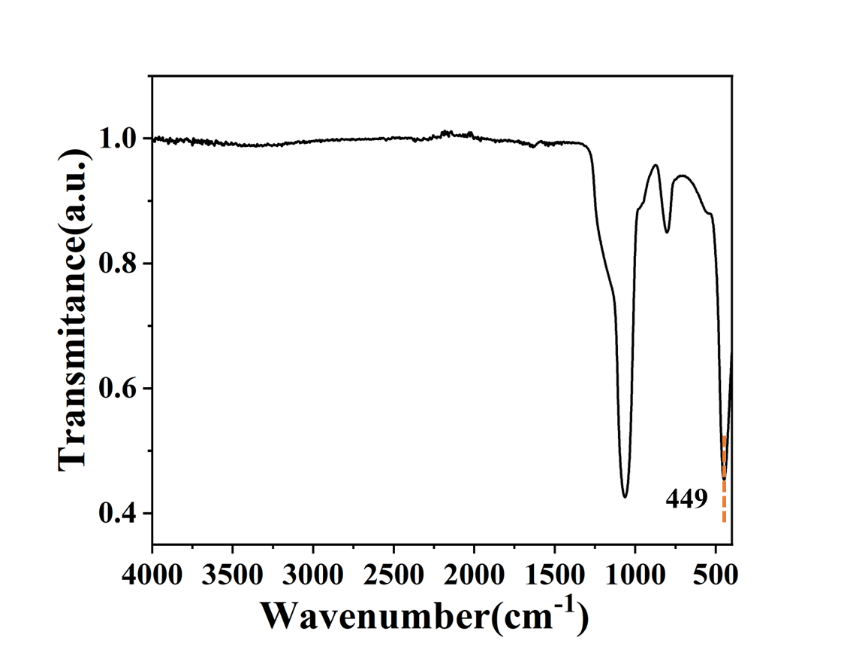
**Figure S3.** FTIR spectra of mesoporous silica nanoparticles in the range of 4000-400 cm^-1^.

The FTIR spectra of mesoporous silica nanoparticles was measured from 4000 to 400 cm^-1^ by FTIR spectrometer. MSNs show clear absorption peaks at 449 cm^-1^ (Figure S3).


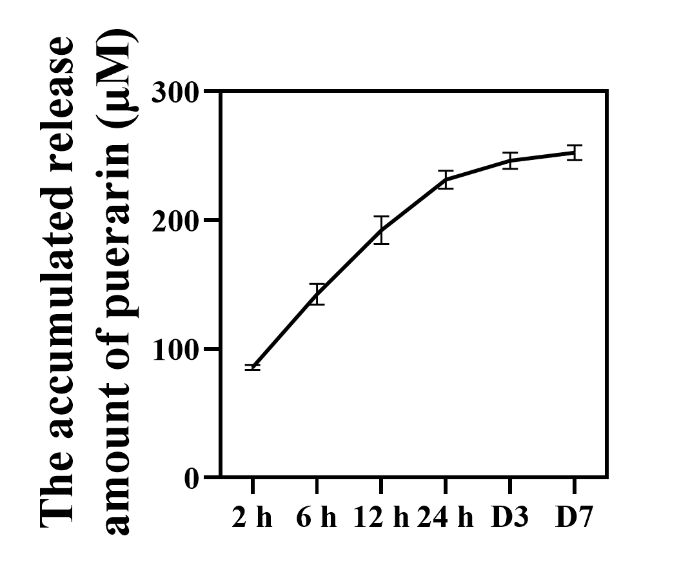
**Figure S4.** The accumulated release amount of puerarin from CHP@Si hydrogel within 7 days.

CHP@Si hydrogel was immersed in PBS and the supernatants were collected by centrifugation at different time points and the content of puerarin in the supernatants was tested. Puerarin in CHP@Si hydrogel show a slow and persistent release behavior (Figure S4).

**Table S1.** Sequence of Primers for qRT-PCR

| Genes | Primer sequences  (F, forward; R, reverse; 5’-3’) | Product length  (bp) |
| --- | --- | --- |
| Mouse TNF**-α** | F: Tta gag cgg gat agt aac g  R: caa aat aca caa cag tgt c | 111 |
| Mouse NF-κB | F: 5' TGA CAA GGT TCA GAA AGA TG 3'  R: 5' GAA GAC AAT GGC AAA CTG 3' | 124 |
| Mouse TGF-β | F: aag gac ctg ggt tgg aag t  R: ggt cct tgc cct cta caa c | 135 |
| Mouse CCR-7 | F: 5' ATC ATC CGT ACC TTG CTC CA 3'  R: 5' CAG GAC CAC CCC ATT GTA G 3' | 117 |
| Human eNOS | F: 5' CCT TCA GTG GCT GGT ACA TG 3'  R: 5' CAG GAT GTT GTA GCG GTG AG 3' | 74 |
| Human FGF-2 | F: 5' TGC TGT TTC TAT GTC GTG GAA 3'  R: 5' AGG CAG TGC TGA TTT TCA GTC 3' | 98 |
